# Local and Regional Breast Cancer Recurrences: Salvage Therapy Options in the New Era of Molecular Subtypes

**DOI:** 10.3389/fonc.2018.00112

**Published:** 2018-04-17

**Authors:** Yazid Belkacemi, Nivin E. Hanna, Clementine Besnard, Soufya Majdoul, Joseph Gligorov

**Affiliations:** ^1^Henri Mondor Breast Center, Radiation Oncology Department of the Henri Mondor University Hospital, University of Paris Est Creteil (UPEC), INSERM Unit 955, EQ07, Créteil, France; ^2^Kasr Al-Aini Center of Clinical Oncology and Nuclear Medicine Department, Cairo University, Cairo, Egypt; ^3^Sorbonne University, INSERM U938, APHP Tenon, Breast Cancer Expert Center, Paris, France

**Keywords:** breast cancer, local recurrence, salvage treatment, radiotherapy, mastectomy, brachytherapy

## Abstract

Isolated local or regional recurrence of breast cancer (BC) leads to an increased risk of metastases and decreased survival. Ipsilateral breast recurrence can occur at the initial tumor bed or in another quadrant of the breast. Depending on tumor patterns and molecular subtypes, the risk and time to onset of metastatic recurrence differs. HER2-positive and triple-negative (TNG) BC have a risk of locoregional relapse between six and eight times than luminal A. Thus, the management of local and locoregional relapses must take into account the prognostic factors for metastatic disease development. It is important to personalize the overall management, including or not systemic treatment according to the metastatic risk. All isolated recurrence cases should be treated with curative intent. Complete surgical resection is recommended whenever possible. Patients who did not receive postoperative irradiation during their initial management should receive full-dose radiotherapy to the chest wall and to the regional lymph nodes if appropriate. Overall, total mastectomy is the “gold standard” among patients who were previously treated by conservative surgery followed by radiation therapy. In terms of systemic therapy, the benefits of additional treatments are not conclusively proven in cases of isolated recurrence. The beneficial role of chemotherapy has been reported in at least one randomized trial, while endocrine therapy and anti-HER2 are common practice. This review will discuss salvage treatment options of local and locoregional recurrences in the new era of BC molecular subtypes.

## Introduction

The treatment of local (LR) and locoregional recurrences (LRRs) of breast cancer (BC) is a multidisciplinary challenge. Data from the literature, including randomized trials, have shown that LRR occur at a rate of 5–15% after conservative surgery or mastectomy and adjuvant radiotherapy (RT) (1–5). The most frequent site of recurrence in the breast is the original quadrant or the chest-wall scar after radical surgery with 60–95% of all LRR (6, 7).

Moreover, the problem of LRR is that it adds an increased risk of simultaneous or delayed systemic dissemination (8, 9). The patient’s survival is significantly reduced within 2 years after salvage therapy with a significant increased risk of metastases (9). However, the latter can depend on several parameters, including molecular subtype of the initial tumor or the metastatic disease (10).

This review will discuss the therapeutic strategy of LR and LRR according to initial treatment and molecular subtype of BC.

## Salvage Therapy for Local Recurrence after Initial Conservative Therapy

The 10-year recurrence rate after conservative treatment is about 10–20% in patients with early stages invasive BC (11). The median time to recurrence, after the end of systemic adjuvant treatment, may be a short period (2–4 years) or significantly prolonged (5–8 years) (11–13). However, many recent publications have shown that these delays may depend on prognostic factors, tumor biology, and molecular subtypes. Thus, luminal A or B cancers relapse two to three times less often than HER2-positive and triple-negative diseases (TNG) (14, 15). Furthermore, the risk of metastatic events in the first 2–3 years is higher in TNG than in other molecular subtypes, rendering it as having the worst prognosis (16).

**Figure 1:**
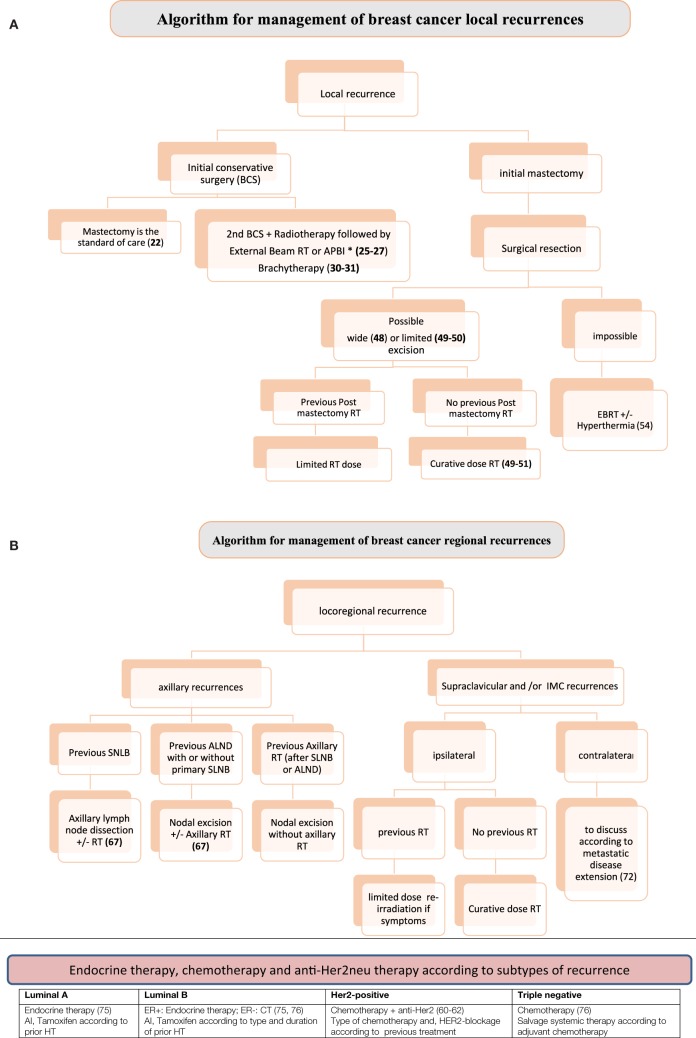
**(A)** Algorithm for management of breast cancer (BC) local recurrences. **(B)** Algorithm for management of BC regional recurrences. Local recurrences: prognosis of TNG and HER2-positive of primary or recurrent BC are mainly driven by the biology of the disease, rather than by the extent of the surgery (17, 51). RT after salvage surgery: HER2 positive are more radioresistant than luminal BC (42, 43) and need more aggressive therapy. Higher metastatic risk in TNG also needs systemic therapy (44, 45). Regional nodal recurrences: after the diagnosis of an LRR, the TNG subtype is associated with a high incidence of distant metastases and cancer-related mortality (66). Systemic therapy has a crucial role after salvage locoregional therapy when indicated. All supraclavicular and IMC recurrences should receive systemic therapy particularly TNG and HER2 positive BC (72–75). Abbreviations: APBI, accelerated partial breast irradiation; RT, radiotherapy; SLNB, sentinel lymph node biopsy; ALND, axillary lymph node dissection; IMC, internal mammary chain.

In cases of isolated LR after conservative surgery, the standard is total mastectomy. However, secondary breast conservation ± RT has to be discussed regarding the benefit/risk ratio and the risk of short-term metastatic dissemination according to tumor biology, such as in TNG patients (14, 15). It is important to note that, although patients with TNG BC have inferior 10-year locoregional outcomes compared with other subtypes (10, 14–16), the data in the literature have no specific surgical implications, because the risk of LRR is unaltered, regardless of whether breast conservation or mastectomy is elected (Figure 1A). This reinforces the concept that the prognosis of TNG and HER2-positive BC are mainly driven by the biology of the disease, rather than by the extent of the surgery (17).

### Salvage Total Mastectomy

According to several guidelines, total mastectomy remains the standard treatment for isolated LR after conservative treatment (18–20). In addition to the indications for total mastectomy, axillary staging is often questioned. Over the past 10 years, many patients had axillary dissection limited to removal of the sentinel lymph nodes. This leaves space for discussing the option of axillary lymph node dissection (ALND) in cases of isolated breast recurrences. Indeed, 31–58% of patients undergoing ALND present histological lymph node involvement (21) which can, in some cases, change the indications for additional systemic therapy. Finally, in the literature, total salvage mastectomy ± ALND allows 85–95% locoregional control (22).

One of the key questions regarding salvage surgery concerns the risk of evolution according to prognostic factors including BC subtypes. In the Canadian study that analyzed the LRR outcomes in patients with stage T1-T2N0 TNG BC who had undergone breast conservation (vs mastectomy) had an absolute reduction in the LRR risk of 6%, after matching for tumor size (23). However, in that study, only limited number of patients had chemotherapy which limits extrapolation of these findings to current practice in which chemotherapy is an essential component of care in TNG and HER2-positive BC.

### Second Breast Conservative Treatment

There is little evidence and limited literature about ipsilateral breast tumor recurrence (IBTR) management by a second conservative surgery ± RT. Thus, in general, it is admitted that secondary breast conservation, when indicated, should include re-irradiation focalized to the site of tumor recurrence only, using external beam radiotherapy (EBRT) or brachytherapy (BCT), while intraoperative approach is under investigation. Outcome of patients after secondary breast conservation may depend on many factors including extend of recurrent disease, quality of salvage surgery and BC subtypes (24).

#### Partial Breast Irradiation Using EBRT Technique

There are no published prospective randomized or ongoing studies that compare mastectomy to a second conservative surgery in patients with isolated LR (22). In the largest retrospective study reported by the Milan team on 134 patients, the 5-year overall survival was 70% in the mastectomy group and 85% after local excision. However, rates of a second LR were higher (19%) after secondary conservative treatment compared with mastectomy (4%) (25). In the series from Kurtz et al. (26), new local relapses after salvage therapy were observed in 31 and 36% of the patients who received or not additional RT. In Deutsch (27) series, the rate of new recurrence was 19% after salvage conservative wide local excision followed by 50 Gy electrons RT. Hormone receptor (HR)-positive patients (62%) received tamoxifen after salvage local treatment. Recently, the RTOG-1014 prospective phase II trial preliminary results were reported at last ASTRO meeting with a subsequent IBTR rate of 3.7% at 3 years. Also, BC subtypes were not analyzed in none of these studies.

#### Partial Breast Irradiation Using BCT

Brachytherapy after second conservative surgery is also an interesting option in the context of prior irradiation because of its ballistic and dosimetric characteristics. The indications for salvage BCT are large with a high rate of relapse preferentially located away from the initial tumor bed (28, 29). In the series from Nice (30) patients who developed LR had a new conservative surgery followed by low dose-rate BCT delivering 45 Gy. After 2 years of follow-up, a new recurrence was observed in 14.5% of patients. Other smaller retrospectives studies, reported similar findings in terms of local control and survival after salvage therapy using BCT (31, 32). None of these studies have analyzed the impact of BC subtypes on outcome.

## Salvage Local Therapy in the Era of BC Molecular Subtypes

Salvage local therapy is mainly defined according to initial treatment including or not adjuvant RT and according to molecular subtype that affect patterns of locoregional and distant recurrences. From RT point of view, the literature is conflicting on BC subtypes radiosensitivity. Recently, Liu et al. (33) have assessed different BC subtypes response to X-ray exposure and found that luminal A diseases did not benefit from RT in contrary to high-risk tumors, such as HER2-positive, basal-like or TNG, even though it is not statistically significant.

The Swedish Breast Cancer Group 91 Radiotherapy trial had demonstrated the importance of RT in all subtypes even for the ER-positive tumors who showed a higher but not significantly different effect (34).

In this context, and without evidence of distant metastases, the discussion of secondary conservative treatment, including BCT or EBRT vs total mastectomy is still a pending question. The delay between initial diagnosis and previous adjuvant treatments has to be considered in the decision. For HER2-positive and TNG recurrences, targeted/systemic therapies are systematically proposed in addition to local salvage therapy. Actually, TNG tumors that express epidermal growth factor receptor or cytokeratin 5/6 demonstrated radioresistance (35).

*In vitro*, clinical trials had also supported radioresistance in HER2-positive tumors and at least three studies had demonstrated that activation of NF-κB and the PI3K/Akt pathway mediate that effect (36–38). Hou et al. (39) described the mechanism of radioresistance to be caused by activation of focal adhesion kinase and epithelial/mesenchymal transition. In fact, Anti-HER2 treatment has been proved to be safe while administered concomitantly with RT, although with careful monitoring of the cardiac condition (40, 41).

In a post-mastectomy setting, the radioresistance of HER2-positive tumors has been also reported (42).

Conversely to HER2-positive and TNG tumors, the results in an adjuvant setting are excellent for luminal tumors with very low rates of recurrences after conservative treatment (Table 1). In case of LR, EBRT or BCT delivered after a second limited excision seems to be less risky. Recently, a randomized clinical trial, conducted by the Swedish group, had evaluated the response of different breast subtypes to adjuvant RT post breast conservation. They concluded that, while HER2-positive tumors seemed to be more radioresistant, luminal tumors (low risk) seem to demonstrate an outstanding effect from RT. In selected cases RT may be an alternative to endocrine therapy (43).

**Table 1:** Recurrence rates according to breast cancer molecular subtypes.

Type	Estrogen receptor	Progesterone receptor	HER2 neu	Ki-67%	Grade	BC subtypes at initial presentation (%) (79)	Recurrence rates	Prognosis
Luminal A	Positive	Positive	Negative	<14	1 or 2	30–40	0.8–8% (14–15–60)	Favorable
Luminal B	Positive	Positive or negative	Negative	>14	2 or 3	20–30	1.5–8.7% (10–59)	Intermediate
Luminal HER2-positive	Positive	Positive or negative	Positive	Any	2 or 3	12–20	1.7–9.4% (14–15–61–63)	Intermediate
HER2 enriched	Negative	Negative	Positive	Any	2 or 3			Unfavorable
Triple-negative	Negative	Negative	Negative	High	Any	15–20	3–17% (64–66)	Unfavorable

In the LR context, salvage surgery followed by RT, seems to be a more adequate option for luminal A compared with HER2-positive or TNG recurrences. After accelerated partial breast irradiation (APBI) using mammosite, a recent study of 1,486 patients showed 5-year IBTR rates of 2.1% for luminal A, 1.5% for luminal B, 4.9% for HER2-positive, and 5.4% for TNG BC. Luminal A and B subtypes compared with the more aggressive HER2 and TNG subtypes combined demonstrated lower 5-year IBTR rates (2.1 vs 5.1%). Moreover, there were higher LRR in TNG patients with lower locoregional control at 5 years (44). Using multicatheter interstitial-APBI, Anderson et al. also showed that molecular subtype influences IBTR and LRR rates. With a longer follow-up (5.4 years), significantly higher IBTR in TNG and HER2-positive BC vs luminal subtypes was noted. More importantly, the regional LNR rates were higher in HER2-positive vs the other subtypes (45). Altogether, these data suggest excluding HER2-positive and TNG BC patients from APBI and also to take into account these results and potential radioresistance for salvage treatments including radiation.

## Salvage Therapy for Local Recurrence after Initial Total Mastectomy

Chest-wall recurrence is associated with a higher risk of metastases more than recurrence after conservative treatment. This risk depends on the delay of appearance of the recurrence, its isolated nature or associated with lymph node recurrence (LNR), number of recurrent nodules, either inflammatory or not, and treatments of recurrence (46). In a recent report of 235 mastectomy patients, the rates of isolated chest wall, regional node and both LRR were 35, 52, and 13%, respectively. This study also showed that an association existed between constructed biologic subtype and median interval time to recurrence following mastectomy. The HER2 and TNG BC patients were shown to have shorter intervals to recurrence (47).

### Surgical Resection

The best local oncological results are obtained by wide excision which must be routinely recommended when possible (48). Limited excision is associated with a second local recurrence in 60–70% of cases (49, 50). Excision of the disease with negative margins increases the chances of subsequent local control. There is a lack of specific data currently regarding the type of salvage surgery indicated, taking into account BC subtypes histology. However, as the risk of nodal involvement is high in TNG and HER2-positive patients, systemic therapy for these subtypes has to be discussed systematically and particularly in patients who did not have previous post-mastectomy RT (PMRT) (50). Initial treatment including or not PMRT is not the only parameter that will impact salvage strategy. There are some consistent data indicating tumor biology impact on outcome. Ursino et al. (51) showed TNG subtype as independent unfavorable prognostic factors either in non-PMRT and PMRT patients, while tumor proliferation was not an unfavorable factor after PMRT.

### Chest-Wall Re-Irradiation

Chest-wall irradiation is the standard of care after wide excision of recurrence when PMRT was not given initially. The curative dose should deliver between 45 and 60 Gy (49, 50). Schwaibold et al. (52) reported that wide excision and RT resulted in a locoregional control rate of 48%. Nevertheless, it seems that there is no dose effect above 50 Gy in cases with free margins. However, indication for additional irradiation seems unavoidable even for small sizes relapse completely resected (53). When radical resection is not feasible RT is an alternative. Its indication depends on the time between first and second RT and pretreatment characteristics. Since the majority of patients receive PMRT as primary treatment, only reduced dose of RT may be delivered. In that case one of the possible options is to combine RT to hyperthermia (HT) to enhance the effects of re-irradiation. One meta-analysis and several retrospective studies have reported higher responses with RT-HT as compared with re-irradiation alone.

Hyperthermia is also considered as an effective option to achieve high local drug concentration in case of unresected disease. Local drug delivery of doxorubicin with thermosensitive liposomes and HT has shown high preclinical therapeutic efficacy in animal models (54). However, drug uptake and chemosensitivity may differ according to the type of BC cell lines models. BC molecular subtypes have not been reported as prognostic factor for HT efficacy after recurrence.

## Salvage Locoregional Therapy in the Era of BC Molecular Subtypes

The development of LNR is a rare event and is usually associated with a poor prognosis. It occurs more commonly in young women with large tumors (55). Outcome of patients with LRR depends on several parameters, mainly of concomitant distant metastases. Rates of distant metastases after LRR are high 57–73% (53, 57). Thus, an aggressive multimodality therapy should be the curative choice for the treatment of patients with isolated LRR without distant metastases (56). One of the key question concerning indications is whether molecular subtypes should be considered for personalized therapy (Figure 1B).

### Impact of BC Molecular Subtypes on LRR

There are many factors that significantly affect the prognosis of patients with LRR. In the report by Schmoor et al. (58), 337 patients developed isolated LRR as the first event after a median follow-up of 8 years. The initial lymph node status, tumor grade, HR status of the primary tumor, and a disease-free interval, at the time of the isolated LRR, have a significant effect on patient outcomes (58).

Luminal A and B subtypes are generally associated with lower risks of regional nodal involvement at diagnosis and tend to have a more indolent evolution as compared with the other subtypes (10, 59). Several retrospective studies have shown lower rates of LRR in luminal A as compared with the other subtypes, among whom luminal B is considered as intermediate risk with rates ranging between 1.5 and 8.7% and peak incidence during the first 5 years (14, 15, 60).

For HER2-positive patients, there are two distinct periods. In studies in which patients were not treated with HER2-targeted therapy, LRR rates were ranged between 4 and 15% (14, 15). More recently, trastuzumab have positively modified the natural course of this BC subtype (10). In a study by Panoff and colleagues (61), patients with HER2-positive tumors who underwent mastectomy and received trastuzumab had LRR rate of 1.7%. This finding was supported by an analysis of six studies by Yin and colleagues (62), who also showed that trastuzumab treatment resulted in a decrease in LRR by 50%. This has been also observed in small tumors HER2-positive BC study after trastuzumab ± chemotherapy (63).

For TNG BC, the involvement of regional lymph nodes is associated with a poor outcome, without a direct relationship to the number of involved nodes (64). In the meta-analysis by Lowery and colleagues (65), the TNG subtype was associated with increased LRR rates, after breast conservation and mastectomy (3–17%). Moreover, after the diagnosis of an LRR, the TNG subtype is associated with a high incidence of distant metastases and cancer-related mortality (66).

### Salvage Therapy According to Nodal Sites of Recurrence and BC Subtypes

While surgical removal of the recurrence is reported to greatly improve survival (67), there are relatively few data concerning regional LNR outcomes in the patients who were treated with a conservative approach. A study by Harris et al. (68) evaluated the risk factors and the prognosis of patients with LNR after breast conservative therapy in stage I–II BC. He concluded that regional LNR may be treated with curative intent. However, it ends up with a poor prognosis as a result of the high risk of simultaneous or subsequent distant metastasis.

According to the 2016 version of the NCCN guidelines, LNR could be treated by salvage locoregional therapy without any distinction according to molecular subtypes. However, when the status at diagnosis is taken into account, Holm-Rasmussen et al. (69) reported from a large cohort of 20,000 patients, that despite the poor prognosis, TNG BC patients have a reduced risk of ALN involvement at the time of diagnosis compared with patients with other subtypes. On the other hand, Liu et al. (70) showed that TNG subtype is not associated more frequently with a higher number of involved nodes. Taken altogether, these data suggest that axillary TNG recurrences should be treated more aggressively including optimal systemic therapy. Local salvage therapy depends on the pervious axilla management (SLNB or ALND ± RT) (Figure 1B).

Supraclavicular nodal recurrence is also predictive of distant metastasis and a poor prognosis. The 5-year distant metastases-free survival is <15% (71). In cases with isolated supraclavicular recurrence, curative systemic and local treatment can be effective. However, in case of contralateral recurrence outcome could be even worse, particularly in non-menopausal patients (72). In general, all BC subtypes of isolated supraclavicular nodal recurrences should be treated definitively by systemic chemotherapy and RT, resulting in outcomes better than previously assumed (73, 74).

Supraclavicular as well as internal mammary (IM) lymph nodes are part of a continuum in the regional lymph drainage of the breast. One large study showed that IM node involvement plays an important role as a prognostic factor for survival (74). Both supraclavicular and IM recurrences are rare, 3–6% for HER2+ and TNG BC (75). The lasts require combined-modality therapy.

Regarding the place of systemic treatment, neither the type of treatments, nor the treatment strategies (neoadjuvant/adjuvant), are clearly defined. Primary systemic treatment is indicated in case of locally advanced disease to enhance the efficacy of locoregional treatments and increase the chance of curability. For “adjuvant” approaches, numerous trials have been published (76–80). Adjuvant endocrine treatment is indicated in case of ER-positive recurrent disease and treatment strategy was validated with tamoxifen (76). However, treatment choice and duration might actually take into account, previous endocrine treatment exposure and if it is the case, disease-free interval to choose between tamoxifen or aromatase inhibitor based therapy. In case of HER2-positive disease and absence of previous anti-HER2 adjuvant treatment, trastuzumab is indicated. However, no one knows what is the optimal strategy in case of previous adjuvant anti-HER2 treatment. According to the results of the CALOR trial, adjuvant chemotherapy should be recommended for patients with completely resected isolated recurrences, especially in ER negative LRR (79). Choice of the drugs, modalities, and duration are not clear, but standard options are usually recommended.

## Conclusion

Local BC recurrence after definitive treatment is a frequently encountered situation in oncology. There are many treatment options, and the validation of their sequence should involve multidisciplinary decision-making. While there is a consensus for therapeutic strategy of LR and LRR according to initial local, the question of personalized systemic therapy taking into account tumor biology and molecular subtypes is still pending. In the TNG and HER2-positive LRR, additional systemic and anti-HER2 therapy seems to be highly indicated. Locoregional RT is usually delivered for all patients who are naïve from RT during initial treatment. However, in case of re-irradiation, discussion of potential radioresistance and expected benefit should guide decisions. Thus, the benefit/risk ratio needs to be explained, the decision validated by the multidisciplinary teams either to tailor the technique to the clinical presentation of recurrence or to personalize systemic therapy.

## Author Contributions

YB and NH wrote the manuscript. CB, SM, and JG completed literature review. CB reviewed and updated all references.

## Conflict of Interest Statement

The authors declare that the research was conducted in the absence of any commercial or financial relationships that could be construed as a potential conflict of interest.
